# Thermal-Ultraviolet-Humidness Coupling Ageing and Regeneration Properties and Mechanisms of SBS-Modified Asphalt Under Hot–Wet Environment Conditions

**DOI:** 10.3390/ma18081731

**Published:** 2025-04-10

**Authors:** Shuo Zhou, Dengfeng Wang, Liuxing Wu, Alimire Maimaitisidike, Zhiqing Wang, Hongbo Zhao, Jiaolong Ren

**Affiliations:** 1 School of Civil Engineering and Geomatics, Shandong University of Technology, Zibo 255000, China; 22110902175@stumail.sdut.edu.cn (S.Z.); 22120908119@stumail.sdut.edu.cn (A.M.); 23507030878@stumail.sdut.edu.cn (Z.W.); hbzhao@sdut.edu.cn (H.Z.); 2 Zhumadian Transportation Construction Technology Center, Zhumadian 463000, China; zs2318976106@163.com; 3 Zhumadian Highway Engineering Development Co., Ltd., Zhumadian 463000, China; 19546325096@163.com

**Keywords:** SBS-modified asphalt, coupling ageing, regeneration, thermal-ultraviolet-humidness, industrial animal oil

## Abstract

Styrene-butadiene-styrene (SBS)-modified asphalt, a widely utilised binder in pavement engineering, is susceptible to ageing due to the coupling effects of thermo-oxidation, ultraviolet radiation, and humidness. Due to the limited availability of high-quality asphalt resources, recycling aged asphalt has emerged as a vital strategy for addressing resource shortages and reducing environmental pollution. This study investigated the effects of thermal-ultraviolet-humidness coupled ageing on the pavement performance of SBS-modified asphalt, with a specific focus on the hot–wet climates of Guangzhou and Chengdu. Beijing’s standard climate serves as a reference for this study. Additionally, industrial animal oil was chosen as a rejuvenator for aged SBS-modified asphalt. The mechanisms underlying hot–wet coupling ageing and regeneration of SBS-modified asphalt were analysed using Fourier Transform Infrared Spectroscopy (FTIR) and Fluorescence Microscopy (FM). The findings indicate that thermal-oxidation and humidness accelerate sulphide formation, resulting in a marked increase in sulfoxide groups and facilitating the migration of lighter components, ultimately leading to asphalt hardening. Under high-temperature and humidness conditions, the butadiene index (BI) of asphalt decreased by 5.96% in Chengdu and 15.78% in Guangzhou compared to Beijing. The sulfoxide index (SI) and aromaticity index (CI) increased by 3.74% and 3.89% in Chengdu, and by 9.39% and 8.54% in Guangzhou, respectively, confirming the exacerbating effect of humidness on ageing. During the regeneration process, industrial animal oil effectively diluted polar molecules in aged asphalt, resulting in reductions in SI by 38.88%, 36.74%, and 37.74%, and in CI by 63.77%, 62.54%, and 63.11% under ageing conditions in Beijing, Guangzhou, and Chengdu, respectively. Rejuvenation is achieved by replenishing lighter components, thereby promoting the aggregation and swelling of the degraded SBS chains.

## 1. Introduction

SBS-modified asphalt is extensively used in highway and urban road construction due to its outstanding high-temperature stability, low-temperature crack resistance, and fatigue resistance [1,2]. However, during service, SBS-modified asphalt pavements undergo ageing reactions induced by the coupling effects of environmental factors, including thermal oxidation, rainfall, and ultraviolet radiation [3,4,5]. These reactions result in a substantial decline in performance, ultimately leading to the material failing to meet service requirements [6,7]. Consequently, examining the ageing characteristics and mechanisms of SBS-modified asphalt under these coupled environmental conditions is essential for the effective construction and maintenance of road traffic infrastructure.

Liu et al. [8] demonstrated that the coupling effects of ultraviolet radiation and temperature accelerate asphalt oxidation. Similarly, Wang et al. [9] and Li et al. [10], through complex modulus and phase angle analyses, found that the interaction between ultraviolet radiation and high temperature degrades the rheological properties of asphalt and shortens its fatigue life. Li et al. [11] observed that the coupling effect of ultraviolet radiation and temperature increased the asphaltene content while decreasing the aromatic content of SBS-modified asphalt. Zhu et al. [12] elucidated the ageing mechanism of SBS-modified asphalt under the coupled influence of ultraviolet radiation and high temperatures using Fourier Transform Infrared Spectroscopy (FTIR). These studies indicate that the ageing effect resulting from the interaction of ultraviolet radiation and temperature is significantly more pronounced than those observed under individual ageing conditions. This highlights the importance of understanding the coupling effects in the real-world service environment of asphalt. Therefore, the influence of humidness, as another critical factor, should also be prioritised, particularly in regions with humid climates [13].

Wei et al. [14] and Zhang et al. [15] found that humidness exacerbates ultraviolet-induced surface cracking in SBS-modified asphalt. Yuan et al. [16] employed microscopy to observe that water infiltrates the inner layers of asphalt through cracks, which weakening the material’s performance, when coupling with high temperatures. Ma et al. [17] confirmed that the humidness-thermal coupling effect disrupts the “bee-like structure” of asphalt, thereby diminishing its bonding properties. Existing research indicates that the ageing process of SBS-modified asphalt is accelerated and intensified under the coupling influence of humidness and high temperatures, suggesting that SBS-modified asphalt pavements are particularly vulnerable to ageing in hot–wet environments [18,19].

However, during its service life, asphalt is influenced not only by dual-factor coupling ageing but also by the coupling effects of thermal-ultraviolet-humidness, which are more likely to accelerate the degradation of asphalt performance. Zou et al. [20] demonstrated that the high-temperature rutting resistance and low-temperature crack resistance of asphalt are significantly diminished after ageing due to thermal oxidation, humidness, and ultraviolet radiation. However, these tests only simulated short-term ageing conditions, and ageing factors were not applied simultaneously. Yang et al. [21] found that the coupling effects of thermal-ultraviolet-humidness significantly intensified microcracks on the surface of SBS-modified asphalt, resulting in performance degradation. Sun et al. [22] demonstrated the mitigating effect of humidness on asphalt ageing, revealing that the water film formed on the asphalt surface reduced the overall impact of the coupled effect of thermal-ultraviolet-humidness. These findings suggest that the coupling effects of thermal-ultraviolet-humidness result in severe asphalt ageing.

However, as a by-product of petroleum [23,24], the availability of high-quality asphalt is decreasing due to the depletion of petroleum resources [25]. Recycling aged SBS-modified asphalt presents an effective and cost-efficient solution to meet the growing demand for asphalt in infrastructure construction. Furthermore, if not properly managed, large quantities of waste SBS-modified asphalt can contribute to environmental pollution and resource depletion [26]. Recycling aged asphalt not only reduces carbon emissions and alleviates environmental pressures but also meets the demand for SBS-modified asphalt in transportation engineering, thereby promoting sustainable resource utilisation [27].

Numerous studies have examined the effects of various recycling technologies on asphalt ageing. Zhang et al. [28] demonstrated that rejuvenators rich in aromatic compounds can effectively restore the performance of aged asphalt by compensating for the loss of lighter components. Wu et al. [29] revealed that rubber hydrocarbon rejuvenators improve the low-temperature crack resistance of ageing SBS-modified asphalt. Zhang et al. [30] demonstrated that rejuvenators rich in lighter components significantly enhanced the high-temperature performance of SBS-modified asphalt. Yao et al. [31] utilised FTIR to elucidate the regeneration mechanism of unaged SBS-modified asphalt when applied to ageing asphalt. Furthermore, bio-oil has been extensively used in asphalt recycling due to its cost-effectiveness and environmental benefits [32,33,34]. Yang et al. [35] validated the feasibility of bio-oil rejuvenators through computational simulations. Li et al. [36] employed FTIR and scanning electron microscopy to elucidate the regeneration mechanism of soybean bio-asphalt. These results indicate that both soybean bio-asphalt and SBS-modified asphalt contain numerous functional groups, which can promote the expansion of SBS to form a network structure. However, existing studies have primarily focused on regeneration under single ageing conditions while neglecting regeneration following thermal-ultraviolet-humidness coupling ageing conditions. Given the substantial impact of multifactor coupling ageing, which causes more severe damage in SBS-modified asphalt, investigating the regeneration properties and mechanisms of SBS-modified asphalt after coupling ageing is of critical importance.

Given that the asphalt performance is significantly influenced by the combined effects of various factors during traffic operations, the objectives of this study are as follows:

- To investigate the thermal-ultraviolet-humidness coupling ageing properties and mechanisms of SBS-modified asphalt under hot–wet environmental conditions.
- To elucidate the regeneration effects and mechanisms of an industrial animal oil rejuvenator on SBS-modified asphalt subjected to coupling ageing.

Therefore, the hot–wet conditions of Guangzhou and Chengdu were selected as research subjects, while the climatic conditions of Beijing served as a control. The thermal-ultraviolet-humidness coupling ageing properties and mechanisms of SBS-modified asphalt were examined under these environmental conditions. Moreover, the coupling aged SBS-modified asphalt was rejuvenated using industrial animal oil, highlighting the effectiveness and underlying mechanisms of the regeneration process. This study not only contributes to the advancement of anti-ageing technologies for SBS-modified asphalt but also promotes the reuse of multifactor coupling ageing.

## 2. Materials and Methods

### 2.1. Materials

**Figure 1:**
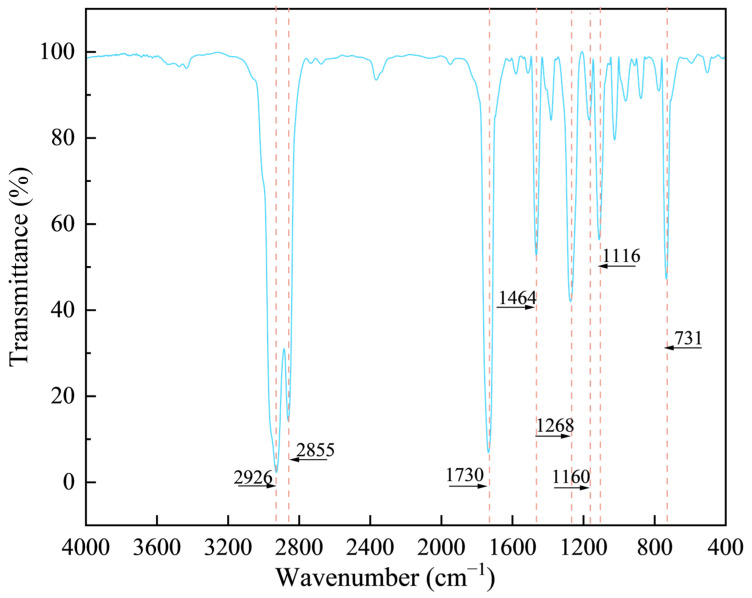
Functional groups in industrial animal oil.

The SBS-modified asphalt produced by Qilu Petrochemical Company (Zibo, China) is manufactured by blending AH-70# base asphalt with 4.5% SBS content relative to the base asphalt, and all key parameters met the specifications “Technical Specifications for Construction of Highway Asphalt Pavements (JTG F40-2004)” [37]. An industrial animal oil produced by Guangzhou Fufei Chemical Technology Co., Ltd. (Guangzhou, China) was selected as the rejuvenator. Table 1 and Table 2 present the key technical parameters of the SBS-modified asphalt and the industrial animal oil, respectively. The functional groups of industrial animal oils were analysed by Fourier transform infrared spectroscopy (FTIR), as illustrated in Figure 1.

As illustrated in the figure, the absorption peaks observed at 2926 cm⁻^1^, 2855 cm⁻^1^, and 1464 cm⁻^1^ correspond to the asymmetric stretching vibration, symmetric stretching vibration, and bending vibration of methylene (CH_2_) groups, respectively. The prominent absorption peak at 1730 cm⁻^1^ is attributed to the stretching vibration of the carbonyl group (C=O), indicating a substantial presence of carbonyl compounds in the industrial animal oil. The absorption peak at 1268 cm⁻^1^ is attributed to the phenolic hydroxyl group, while the peak at 1116 cm⁻^1^ corresponds to the stretching vibration of ether linkages (C-O-C) within lipid chains. Additionally, the prominent absorption peak at 731 cm⁻^1^ is associated with the out-of-plane bending vibration of aromatic carbon-hydrogen (C-H) groups in benzene rings, indicating the presence of aromatic compounds in industrial animal oil.

### 2.2. Experimental Methods

#### 2.2.1. Ageing Process

**Figure 2:**
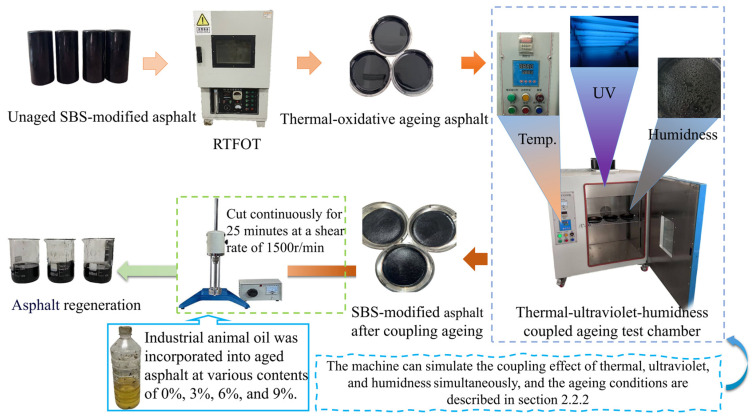
Experimental flow chart.

According to the Chinese test standard “Standard Test Methods of Bitumen and Bituminous Mixtures for Highway Engineering (JTG E20-2011) [38]”, the Rolling Thin Film Oven Test (RTFOT) is utilised to conduct thermal-oxidative ageing on asphalt binders. Each test specimen is prepared with a mass of 35 ± 0.5 g and subjected to thermal ageing at 163 °C ± 0.5 °C for a duration of 85 min. Subsequently, the thermal-oxidative ageing asphalt is placed in a coupling ageing test chamber, where it undergoes the coupling action of thermal oxidation, ultraviolet radiation, and humidness. The parameters for each ageing condition are detailed in Section 2.2.2. The aged asphalt is then mixed with varying contents of industrial animal oil (0%, 3%, 6%, and 9%) for a continuous duration of 25 min at 1500 r/min in a shearing unit, ensuring thorough integration of the industrial animal oil with the aged asphalt. The detailed process is illustrated in Figure 2.

#### 2.2.2. Ageing Conditions

To accurately simulate the asphalt ageing environments in Beijing, Guangzhou, and Chengdu, the specific experimental conditions present in each city were analysed. Given that asphalt ageing is most pronounced during the summer, the summer climatic characteristics of these three cities were used as benchmarks to design experimental groups incorporating distinct ageing factors. A thermal-ultraviolet-humidness coupling ageing chamber was employed to simulate the ageing processes of asphalt under controlled laboratory conditions, effectively replicating real-world ageing phenomena.

(1) Based on meteorological data from the China Meteorological Data Network [39], a statistical analysis was conducted on the total summer rainfall in Beijing, Guangzhou, and Chengdu from 2001 to 2020, and the average daily rainfall was calculated accordingly. Detailed data are presented in Table 3.

(2) According to the “Technical Specifications for Construction of Highway Asphalt Pavements (JTG F40-2004)” [37], Beijing is classified as a hot summer and cold winter climatic zone, while Chengdu is categorised as a warm-winter climatic zone. In all three cities, the average maximum temperature during the hottest month exceeds 30 °C, with temperatures of 35 °C and above being more frequent in Guangzhou. Consequently, the average summer temperatures in Beijing, Guangzhou, and Chengdu are 30 °C, 35 °C, and 30 °C, respectively. Generally, the surface temperature of asphalt pavement is approximately 20 to 35 °C higher than the ambient air temperature. Therefore, experimental temperatures of 65 °C, 70 °C, and 65 °C were chosen as the ageing environmental conditions for these three cities.

(3) This study utilised a thermal-ultraviolet-humidness coupling ageing chamber equipped with six 40 W ultraviolet lamps. The summer solar radiation levels in Beijing, Guangzhou, and Chengdu were determined using solar radiation data from the China Meteorological Data Network, with ultraviolet radiation accounting for approximately 5% to 7% of the total solar radiation. Based on the energy equivalence, the ultraviolet radiation exposure time for the three cities were calculated using Equation (1). Specific data are presented in Table 4.

(1) $$T=\frac{Y}{3600\cdot X}$$

where *T* represents the indoor ultraviolet irradiation duration (h); *X* denotes the irradiation intensity of the ultraviolet lamps (W/m^2^); and *Y* corresponds to the average summer solar ultraviolet irradiation intensity (MJ/m^2^).

Based on the aforementioned findings, the experimental coupled ageing conditions were categorised as follows: Beijing served as the benchmark environmental conditions (BEC), Guangzhou represented a high-temperature and high-humidness environment conditions (HEC), and Chengdu was classified as a slightly high-temperature and high-humidness environmental conditions (SHEC). Detailed parameters are illustrated in Table 5.

#### 2.2.3. Test Methods

The high and low temperature properties of asphalt after ageing and regeneration were evaluated using the softening point test and the 5 °C ductility test, respectively. According to the Chinese test standard “Standard Test Methods of Bitumen and Bituminous Mixtures for Highway Engineering (JTG E20-2011) [38]”, two specimens are measured for the softening point, and three specimens are used for the 5 °C ductility.

A Nicolet 5700 Fourier transform infrared spectrometer (Waltham, MA, USA) and an LW100 FT/B fluorescence microscope (Cossim, Beijing, China) were employed to analyse the microstructure of the asphalt, investigate the chemical functional groups present in aged and recycled asphalt, and evaluate the size and dispersion of the SBS. The infrared spectrum of the asphalt material ranged from 400 cm⁻^1^ to 4000 cm⁻^1^, with a resolution of 0.4 cm⁻^1^. The fluorescence microscope provided a magnification of 400×.

## 3. Pavement Properties

### 3.1. Pavement Properties After Ageing

**Figure 3:**
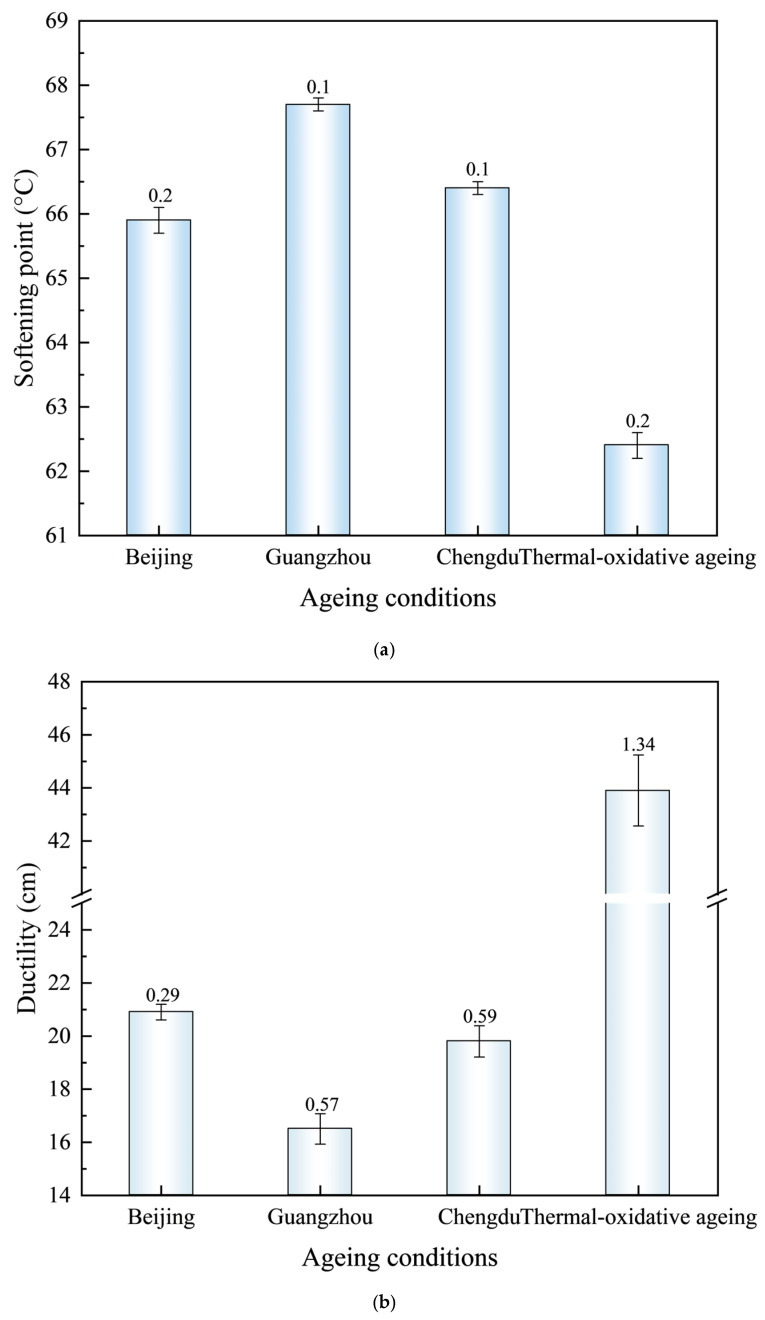
The performance of SBS-modified asphalt pavement after ageing. (**a**) Softening point. (**b**) Ductility.

The softening point and ductility of SBS-modified asphalt under different ageing conditions were tested, and the results are illustrated in Table 6 and Figure 3.

Under coupled ageing conditions, the performance of the SBS-modified asphalt undergoes significant changes. As illustrated in Figure 3, the softening point and ductility of the SBS-modified asphalt exhibited varying degradation patterns under three distinct environmental conditions: Guangzhou, Chengdu, and Beijing. Thermal-oxidative ageing results in a 24.36% reduction in the softening point and a 28.50% decrease in ductility, indicating a significant deterioration in high-temperature performance due to the coupled effects of thermal-oxidative ageing. Following thermal-ultraviolet-humidness coupling ageing, the softening points under the BEC, HEC, and SHEC increased by 5.61%, 8.49%, and 6.41%, respectively, compared to those observed after thermal-oxidative ageing. In contrast, the ductility values decreased by 52.39%, 62.41%, and 54.90% under the BEC, HEC, and SHEC, respectively. The degree of ageing among the three cities can be ranked as follows: HEC > SHEC > BEC. Specifically, compared to BEC in Beijing, the HEC in Guangzhou exhibited a 2.73% increase in the softening point, but a 21.05% greater reduction in ductility. Similarly, the SHEC in Chengdu showed a 0.76% increase in softening point, accompanied by a 5.26% decrease in ductility. This phenomenon can be attributed to variations in the environmental parameters. The humidness and ultraviolet radiation levels in the HEC are 2.55 times and 0.72 times higher, respectively, than those in the BEC, with a temperature that is 5 °C higher. The SHEC in Chengdu exhibits humidness and ultraviolet radiation levels that are 2.0 times and 0.59 times higher, respectively, than those recorded at BEC under comparable temperature conditions. Notably, although the BEC in Beijing experiences the most intense ultraviolet radiation, the severity of ageing is lower than that observed under HEC. This finding that asphalt ageing results from the synergistic interactions among thermal, ultraviolet, and humidness factors. Moreover, the coupled high-temperature and high-humidness conditions in the HEC environment induce significantly more severe ageing effects compared to the environmental conditions in BEC. This observation further confirms that ultraviolet radiation does not play a dominant role in the ageing process of SBS-modified asphalt. Furthermore, although the high-temperature and humidness environment conditions in Chengdu present lower ultraviolet conditions compared to the benchmark conditions in Beijing, their increased humidness suggests that the coupling of humidness and ultraviolet radiation amplifies the synergistic ageing mechanism involving thermal, ultraviolet exposure, and humidness.

### 3.2. Pavement Properties After Regeneration

**Figure 4:**
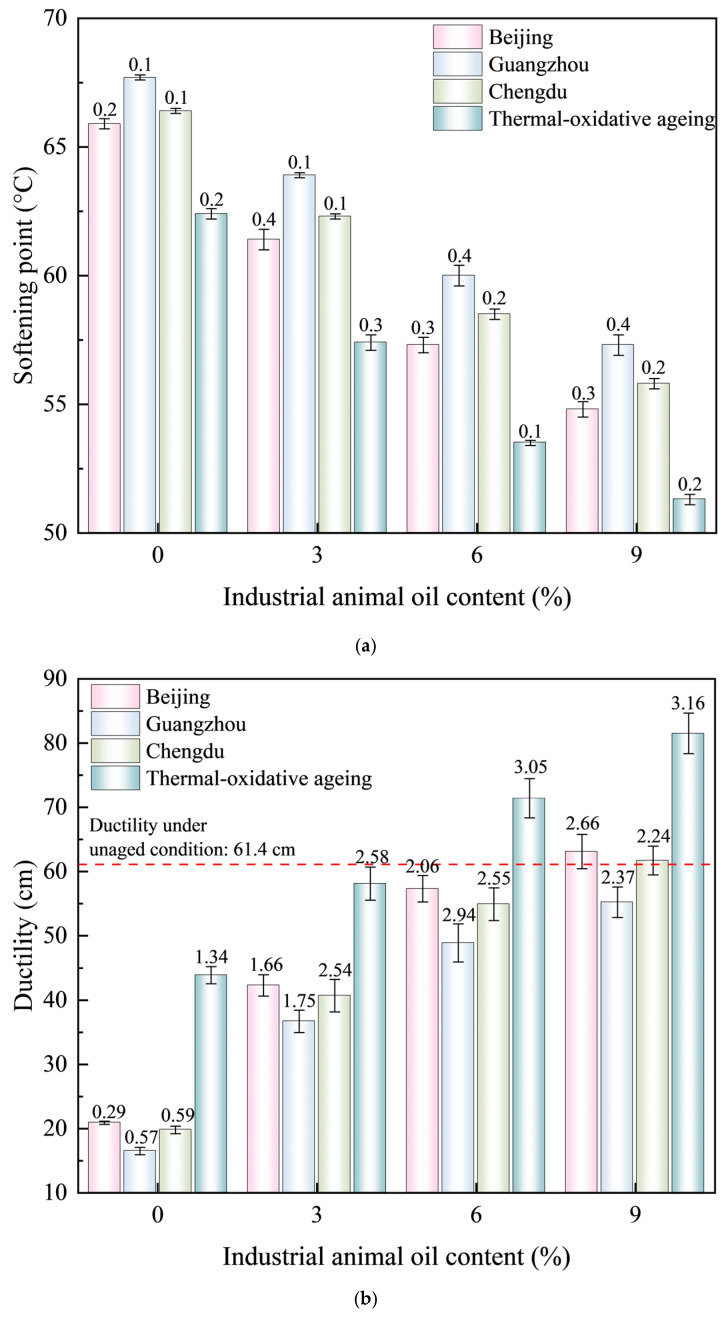
Road performance of SBS-modified asphalt at varying modifier contents. (**a**) Softening point. (**b**) Ductility.

Softening point and ductility tests were conducted on recycled asphalt with varying additive contents of 0%, 3%, 6%, and 9%. The results are presented in Table 7 and Figure 4.

As illustrated in Figure 4, the softening point of the SBS-modified asphalt decreases notably with increasing concentrations of industrial animal oil, while its ductility is significant enhanced. This observation indicates that the high-temperature performance of the SBS-modified asphalt does not improve during the regeneration process; however, its stress-bearing capacity under low-temperature conditions is markedly enhanced. When 3% industrial animal oil was added, the ductility of the BEC-, HEC-, SHEC-aged, and thermal-oxidative SBS-modified asphalts increased by 102.39%, 118.79%, 105.55%, and 32.35%, respectively, compared to the aged SBS- modified asphalt. Conversely, the softening points decrease by 6.82%, 5.61%, 6.17%, and 8.01%, respectively. The three types of coupled aged asphalt exhibited significant improvements in ductility, with the thermal-oxidative aged asphalt showing the smallest increase in ductility. In terms of the reduction in the softening point, the thermal-oxidative aged asphalt experienced the most pronounced decrease, whereas HEC-aged asphalt exhibited the least reduction. These findings indicate that the addition of a low content of industrial animal oil effectively replenishes the components lost by the asphalt during coupled ageing, thereby significantly enhancing its low-temperature performance. When the industrial animal oil content reached 6%, the ductility of the thermal-oxidative aged asphalt was 1.16 times greater than that of the unaged asphalt. In this scenario, the ductility of the BEC and SHEC-aged asphalts recovered to 93.32% and 89.41% of their unaged states, respectively, while the HEC-aged asphalts recovered only 78.66% of its unaged state. When the industrial animal oil content was further increased to 9%, the ductility of the BEC and SHEC-aged asphalt surpassed that of the unaged asphalt, whereas the HEC-aged asphalt recovered only 88.93% of the ductility of the unaged asphalt. Concurrently, the softening points were reduced to 66.42%, 67.64%, and 69.45% of the unaged asphalt. The recovery performance of the HEC-aged indicates that thermal-ultraviolet-humidness coupling effects under high-temperature and high-humidness conditions induce significant ageing damage, leading to significant degradation in low-temperature performance, which is challenging to restore. In contrast, the decline in the high-temperature performance was partially mitigated by the increased hardness of the asphalt.

**Figure 5:**
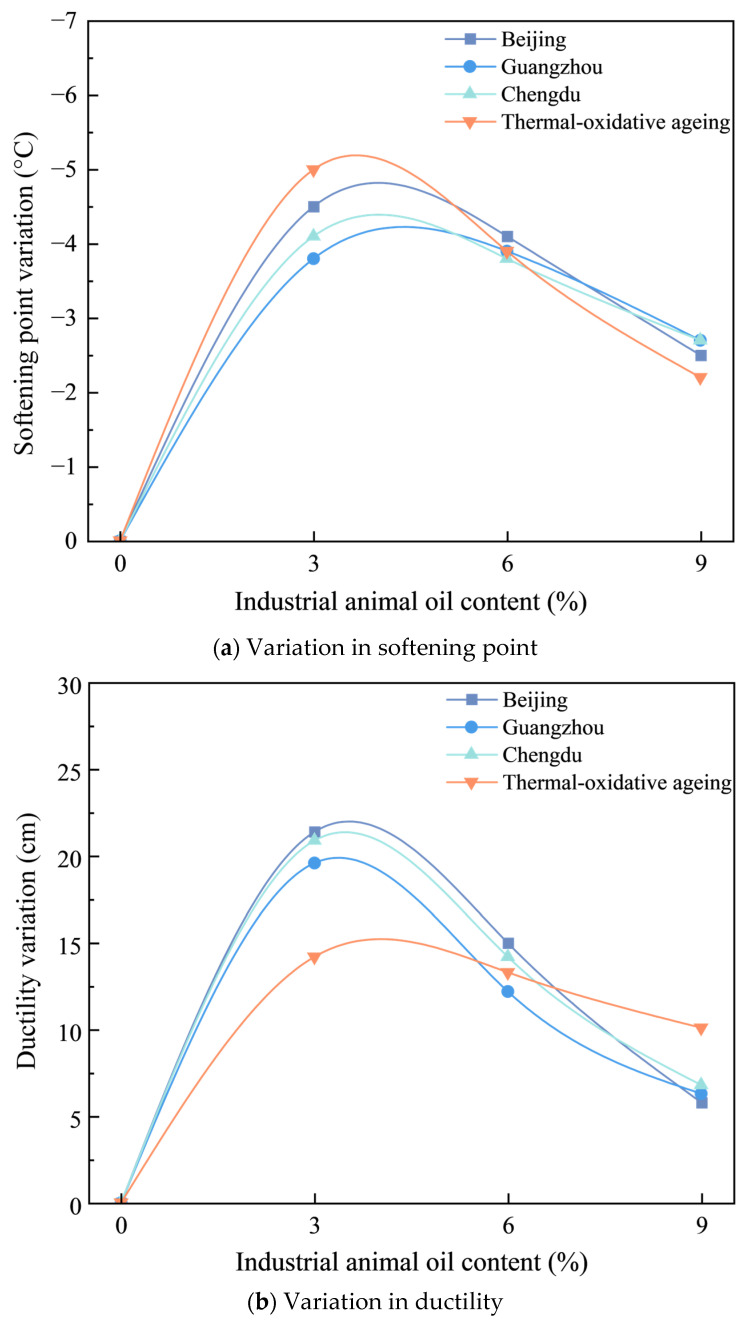
Variation in softening point (**a**) and ductility (**b**) with varying amounts of industrial animal oil.

In order to further investigate the regenerative effects of industrial animal oil at varying contents, we calculated the changes in softening point and ductility under different ageing conditions. The results are presented in Figure 5.

As illustrated in Figure 5, the addition of industrial animal oil to aged asphalt results in both increases and decreases in the softening point and ductility. Initially, these changes increase and then decrease, with the maximum change occurring at an industrial animal oil content of approximately 4%. This indicates that the restorative effect of industrial animal oil on aged asphalt initially enhances its properties before gradually diminishing. The addition of industrial animal oil can supplement essential components in asphalt, thereby improving the performance of aged asphalt and facilitating its restoration. However, as the integration of industrial animal oil approaches saturation, the restorative effect also declines. Notably, as the content of industrial animal oil increases, the absolute value of the softening point shows a downward trend, while the absolute value of ductility exhibits an upward trend.

In addition, as illustrated in Figure 5a, when the content of industrial animal oil is less than 6%, the reduction in the softening point of asphalt under thermal-oxidative ageing conditions is greatest, while the reduction observed in Guangzhou is the smallest. However, as the content of industrial animal oil continues to increase, the softening point of aged asphalt under thermal-oxidative ageing conditions decreases more rapidly, falling below the levels observed under coupled ageing conditions. As illustrated in Figure 5b, when the content of industrial animal oil is less than 6%, the increase in the ductility of asphalt under thermal-oxidative ageing conditions is minimal and significantly lower than that of asphalt subjected to coupled ageing conditions. However, as the content of industrial animal oil continues to increase, the ductility of aged asphalt under thermal-oxidative ageing conditions improves more significantly than under coupled ageing conditions. This indicates that, when the content of industrial animal oil reaches a certain level, it becomes easier for the asphalt to reach saturation under coupled ageing conditions.

## 4. Ageing and Regeneration Mechanisms

### 4.1. Functional Group

**Figure 6:**
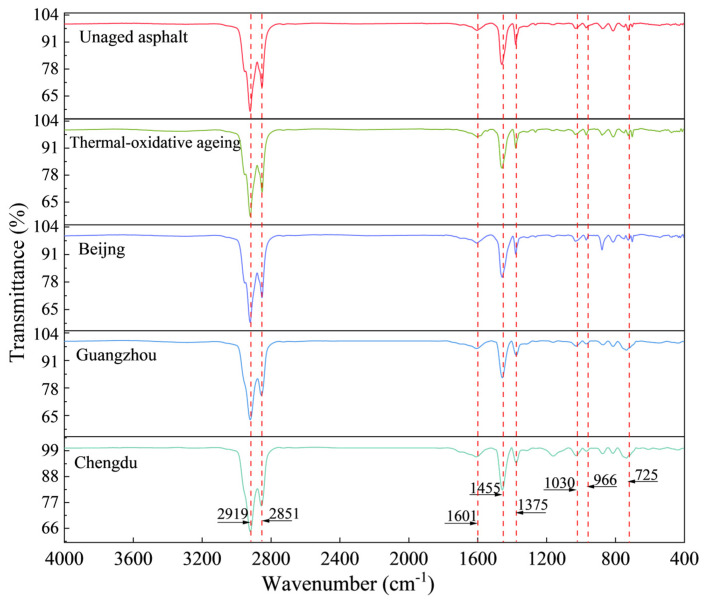
Functional groups of SBS-modified asphalt under different ageing conditions.

Figure 6 illustrates the evolution of functional groups in SBS-modified asphalt under various ageing conditions, as analysed by FTIR spectroscopy.

**Figure 7:**
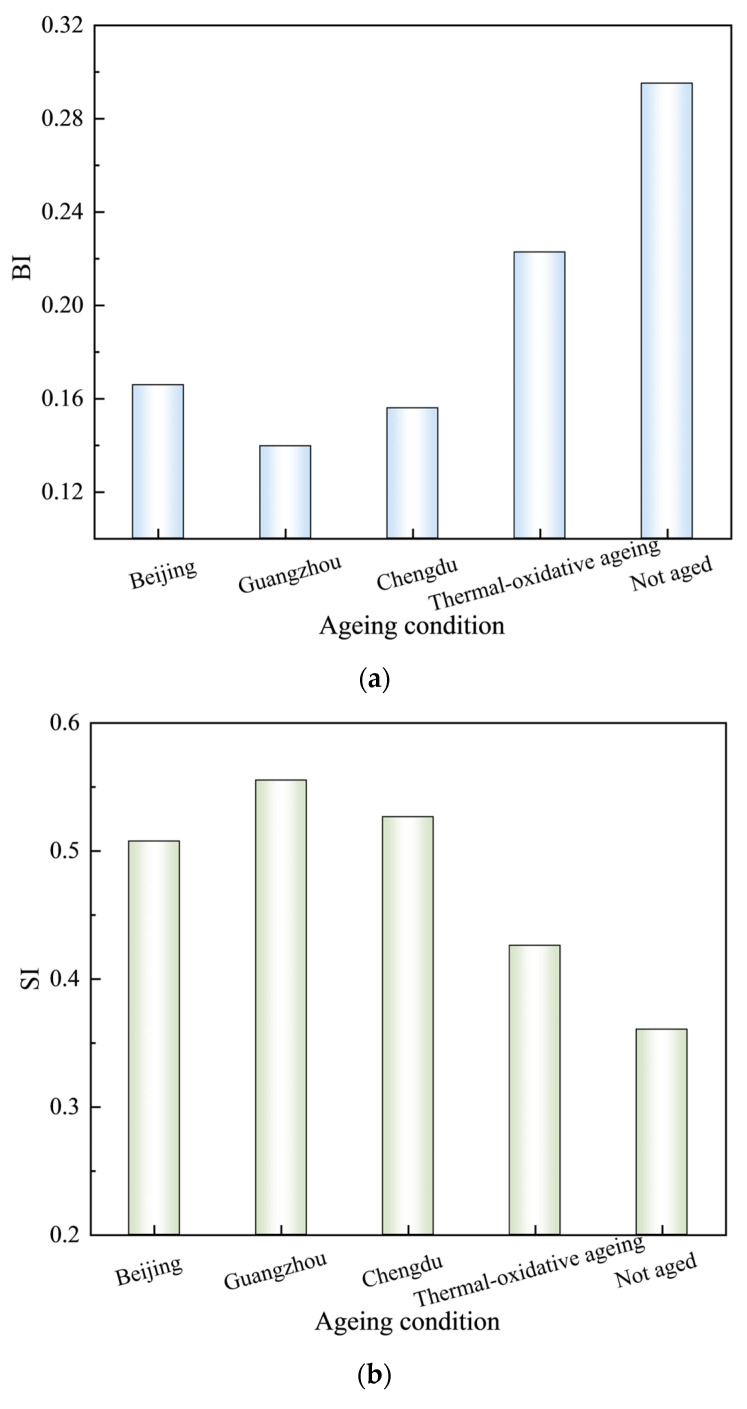

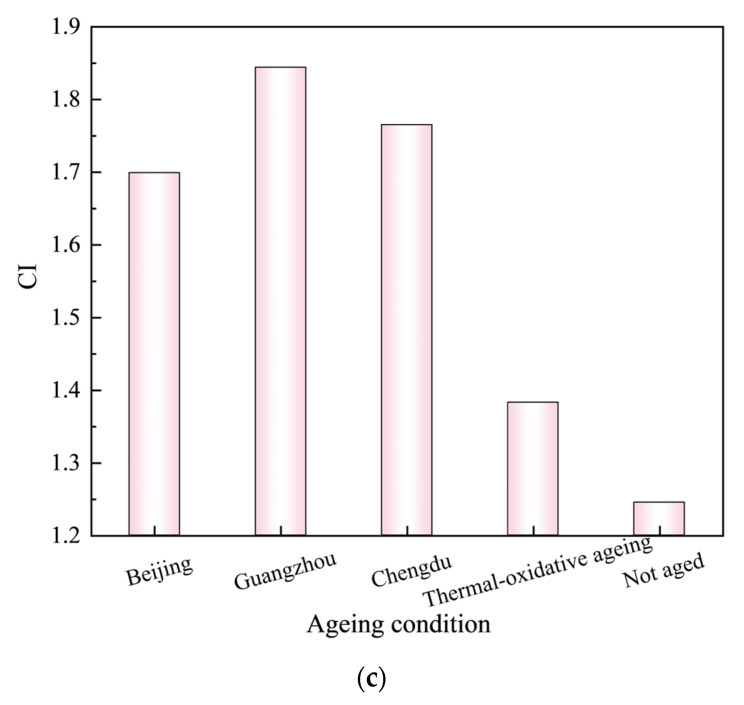
BI, SI, and CI under various ageing conditions. (**a**) BI after ageing. (**b**) SI after ageing. (**c**) CI after ageing.

As illustrated in Figure 6, distinct absorption peaks are evident in the FTIR spectra of asphalt subjected to various ageing conditions at 2919 cm⁻^1^, 2851 cm⁻^1^, 1601 cm⁻^1^, 1455 cm⁻^1^, 1375 cm⁻^1^, 1030 cm⁻^1^, 966 cm⁻^1^, and 725 cm⁻^1^. The strong peaks at 2919 cm⁻^1^ and 2851 cm⁻^1^ are attributed to the asymmetric and symmetric stretching vibrations of C-H bonds in methylene (-CH_2_-) groups, respectively. Additionally, the absorption peaks detected at 1601 cm⁻^1^, 1455 cm⁻^1^, 1375 cm⁻^1^, and 1030 cm⁻^1^ are assigned to the stretching vibrations of C=C bonds with in condensed aromatic rings, the asymmetric bending vibrations of methyl (-CH_3_) groups, the symmetric bending vibrations of methyl (-CH_3_) groups, and the stretching vibrations of sulfoxide (S=O) functional groups, respectively. Notably, the peaks at 966 cm⁻^1^ and 725 cm⁻^1^ are attributed to the bending vibrations of C=C bonds in polybutadiene and the wagging vibrations of methylene (-CH_2_-) groups in the aromatic rings of polystyrene, respectively, serving as characteristic absorption peaks for SBS modifiers. The intensity of the absorption peaks varied, while their positions remained relatively stable. This indicates that the types of functional groups in the asphalt did not change, only their concentrations did. Under different ageing conditions, the decrease in intensity at 966 cm⁻^1^ indicated the scission or crosslinking of butadiene within the asphalt. Meanwhile, changes in peak intensity at 1030 cm⁻^1^ and 1600 cm⁻^1^ indicate an increased degree of oxidation and enhanced aromatic conjugation, respectively. Previous studies have established a correlation between the areas of these absorption peaks and the concentrations of the respective functional groups [40]. Given the relatively small absorption peak area of carbonyl groups, which can introduce significant measurement errors, this study employs three indices to characterise the ageing and regeneration mechanisms of SBS-modified asphalt: the Butadiene Index ($BI=A_{966}/A_{1375}$), which quantifies SBS content; the Sulfoxide Index ($SI=A_{1030}/A_{1375}$), which measures S=O groups; and the Aromaticity Index ($CI=A_{1600}/A_{1375}$), which assesses C=C bonds. $A_{966}$, $A_{1030}$, $A_{1600}$, and $A_{1375}$ represent the absorption peak areas at 966 cm⁻^1^, 1030 cm⁻^1^, 1600 cm⁻^1^, and 1375 cm⁻^1^, respectively. The calculated values of BI, SI, and CI under various ageing conditions are presented in Table 8 and Figure 7.

As illustrated in Figure 7, the asphalt subjected to various ageing conditions exhibited notable changes in its chemical properties compared to the unaged asphalt. Specifically, the butadiene index (BI) of the aged asphalt decreased significantly, while the sulfoxide index (SI) and aromaticity index (CI) showed increasing trends. These findings suggest a reduction in the concentration of butadiene functional groups in the SBS-modified asphalt during the ageing process, accompanied by an increase in sulfoxide and oxygen-containing groups, as well as aromatic components. This observation highlights the molecular degradation of the SBS polymers and the progression of oxidation reactions during asphalt ageing. Thermal-oxidative ageing resulted in a 24.56% decrease in the BI, an 18.18% increase in the SI, and an 11.06% increase in the CI. These changes provide clear evidence of the pronounced oxidation reactions occurring in asphalt under thermal-oxidative conditions. The substantial decrease in the BI was accompanied by a marked reduction in the softening point, as discussed in Section 3.1. The migration of lighter components during this process is insufficient to mitigate the deterioration of asphalt’s high-temperature performance, resulting in a pronounced decline in its high-temperature characteristics. Following thermal-ultraviolet-humidness coupling ageing treatment, BEC, HEC, and SHEC exhibited additional BI reductions of 25.57%, 37.31%, and 30.00%, respectively, compared to thermal-oxidative ageing. Furthermore, increases in SI of 19.17%, 30.35%, and 23.62% were observed, along with increases in cohesion CI of 22.85%, 33.35%, and 27.63%, respectively. The severity of butadiene degradation and the increase in sulfoxide and aromatic groups followed the order: high temperature and humidness environment conditions > slightly high temperature and humidness environment conditions > benchmark environment conditions. This trend aligns with the ageing intensity hierarchy established in Section 3.1. These findings indicate that thermal-ultraviolet-humidness coupling ageing predominantly accelerates the thermal-oxidative degradation of butadiene, leading to the disruption of the cross-linked network structure within the asphalt. Concurrently, this process promotes the oxidation of thioether compounds into sulfoxide groups, enhancing intermolecular interactions and inducing the migration of lighter components, As a result, the hardness of the asphalt increases. Consequently, these chemical transformations lead to elevated softening points and reduced ductility in asphalt performance metrics.

A comparative analysis between HEC and BEC revealed that HEC exhibited a 15.78% lower BI, 9.39% higher SI, and 8.54% higher CI. These results indicate that the combined effects of Guangzhou have a greater impact on asphalt ageing than Beijing, which are characterised by high ultraviolet radiation. A high-temperature and high-humidness environment accelerates the degradation of butadiene and the oxidation reactions within the asphalt matrix. Compared to the findings of Ma et al. [41], who demonstrated the prompting effects of a coupled humidness and thermal environment on asphalt ageing, the above results indicate that a similarly detrimental effect is also exerted by highly humidness and thermal conditions present in coupled thermal-ultraviolet-humidness ageing. A comparison between SHEC and BEC revealed that, SHEC exhibited a 5.96% decrease in BI, accompanied by increases of 3.74% and 3.89% in SI and CI, respectively. These findings indicate that Chengdu exert stronger coupling effects than Beijing, thereby confirming the role of humidness in accelerating the degradation and oxidation of butadiene. Mechanistically, under high-humidness conditions, the temperature-induced formation of abundant polar groups within the asphalt leads to the formation of hydrogen bonds with water molecules. This interaction facilitates molecular migration within the asphalt and intensifies oxidation reactions. Notably, the ageing of asphalt in Beijing was constrained by the surface hardening of the aged materials, which impeded the penetration of ultraviolet radiation into the deeper layers. However, this constraint had a limited effect on the coupling interactions between temperature and humidness, as liquid water or temperature-induced water vapour could still diffuse into the interior of the asphalt, disrupting its colloidal structures. Collectively, these findings underscore that the coupled influence of temperature and humidness exerts a more substantial effect on butadiene degradation and oxidation than ultraviolet radiation, with elevated humidness levels significantly enhancing the ageing mechanisms.

**Figure 8:**
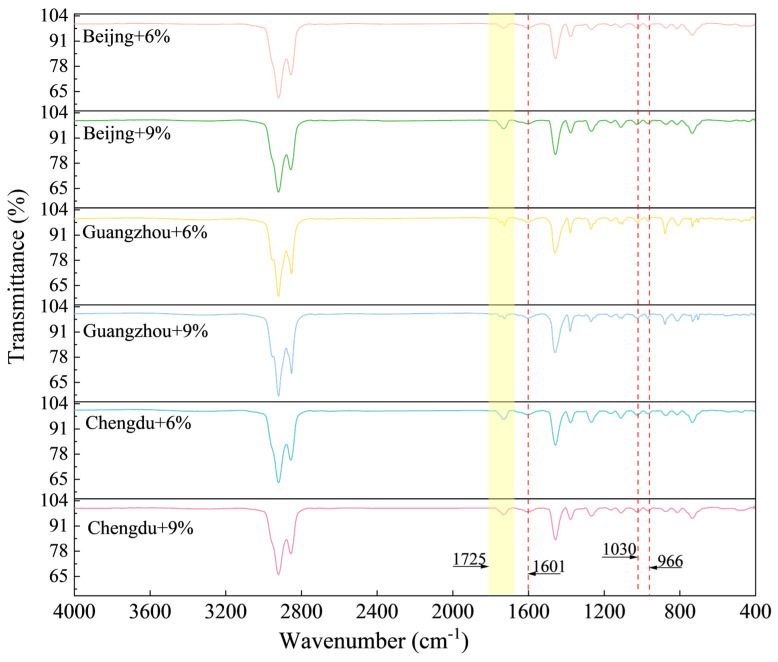
Regenerated functional groups of aged asphalt with 6% and 9% industrial animal oil contents.

**Figure 9:**
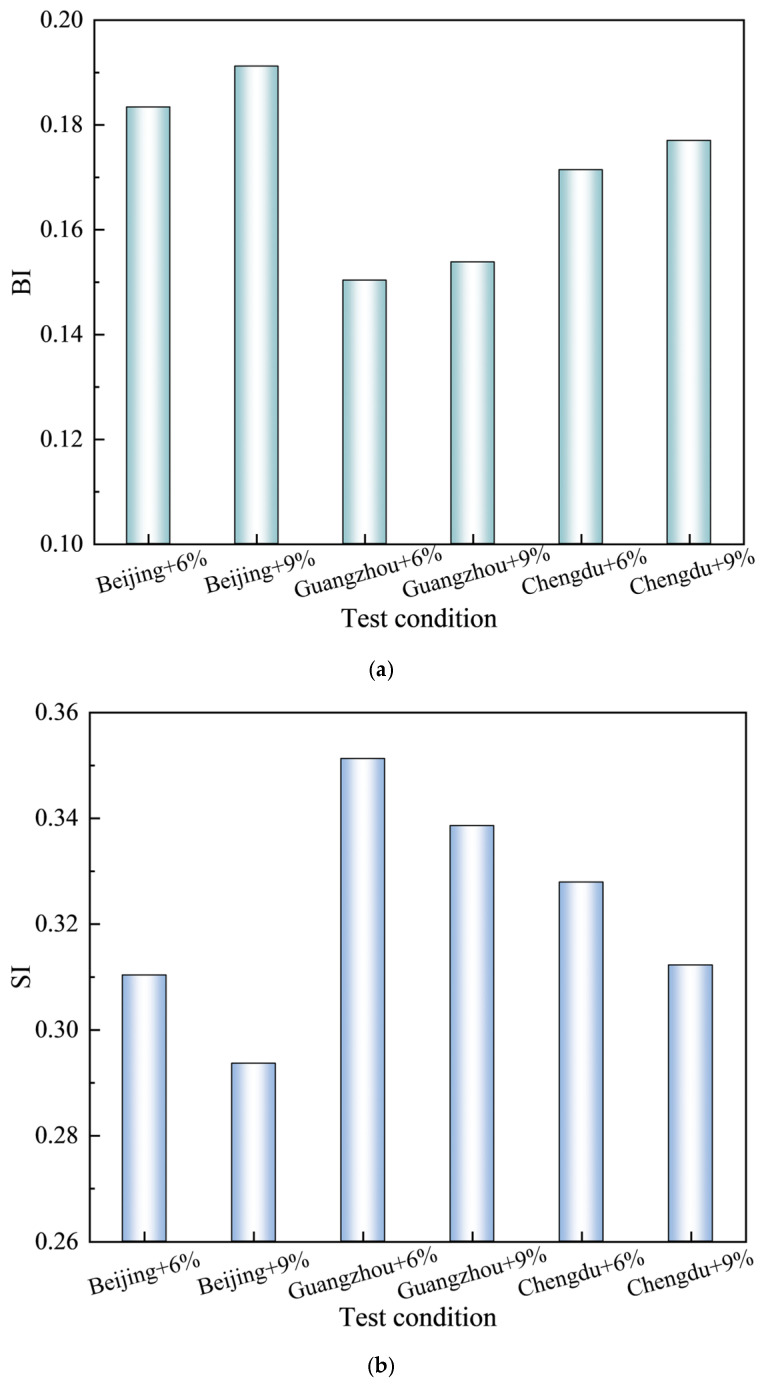

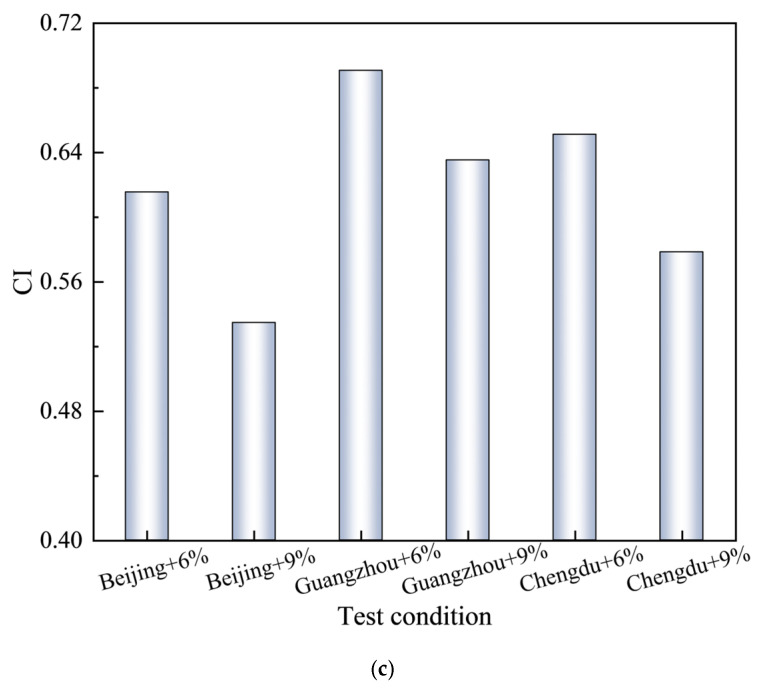
BI, SI, and CI values for samples from Beijing, Guangzhou, and Chengdu with 6% and 9% industrial animal oil contents. (**a**) BI after regeneration. (**b**) SI after regeneration. (**c**) CI after regeneration.

FTIR analysis of rejuvenated asphalt was conducted to calculate the corresponding BI, SI, and CI indices, as illustrated in Figure 8, Table 9, and Figure 9, respectively.

As illustrated in Figure 8, a new peak emerges at 1725 cm⁻^1^, indicating chemical interactions between industrial animal oil and aged asphalt. This peak was attributed to the stretching vibration of the carbonyl groups (C=O) present in the fatty acids of industrial animal oils. Figure 9 illustrates that, with increasing industrial animal oil content, all groups exhibited significant upward trends in BI, accompanied by notable reductions in SI and CI, consistent with the observed variations, aligning in pavement properties. This indicates that the incorporation of industrial animal oil not only increases the butadiene content but also decreases the concentrations of both polar and non-polar functional groups, further validating its effectiveness in rejuvenation. Compared to asphalt subjected to coupling ageing, the incorporation of a 6% content of industrial animal oil resulted in increases in the BI of 10.64%, 7.66%, and 9.96% for BEC, HEC, and SHEC, respectively. This modification was accompanied by reductions in the SI by 38.88%, 36.74%, and 37.74%, and decreases in the CI by 63.77%, 62.54%, and 63.11%, respectively. The relatively modest improvements in BI suggest that the restorative effects of industrial animal oil on SBS polymers are limited. In contrast, the significant reductions in SI and CI indicate that the primary rejuvenation mechanism involves the dissolution and dilution of both polar and non-polar molecules generated during the ageing process, along with the replenishment of lighter components lost over time. When the concentration of industrial animal oil was increased to 9%, the BI, SI, and CI values initially exhibited slight increases before subsequently decreasing under all ageing conditions. Although these indices approach the levels observed in the unaged asphalt, they remain below the benchmarks established for unaged materials. This observation suggests that, while higher contents of industrial animal oil can further enhance asphalt performance, the overall efficacy of the rejuvenation process remains limited. Specifically, the lower index levels observed in SHEC and HEC at a 9% content indicate an intensified interaction between humidness and other ageing factors under their respective environmental conditions. The persistent performance gap between regenerated asphalt and unaged asphalt underscores the inherent limitations of industrial animal oil in fully restoring aged asphalt to its original state, particularly in response to complex ageing mechanisms driven by interrelated environmental factors.

### 4.2. Microscopic Morphology

**Figure 10:**
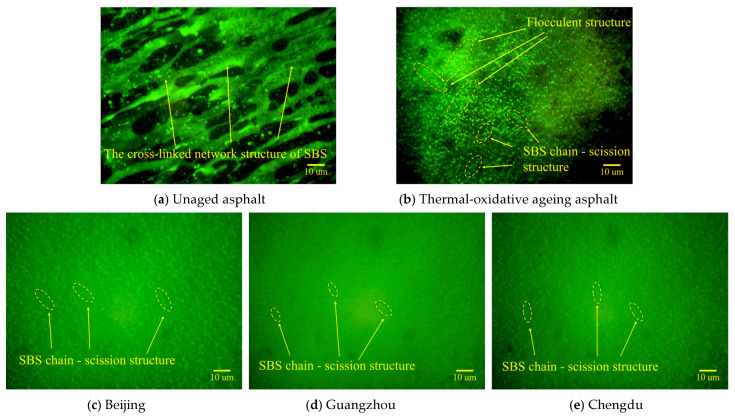
Morphology and distribution of SBS-modifiers in: (**a**) unaged asphalt, (**b**) thermal-oxidative ageing asphalt, and coupling-aged asphalts from (**c**) Beijing, (**d**) Guangzhou, and (**e**) Chengdu.

**Figure 11:**
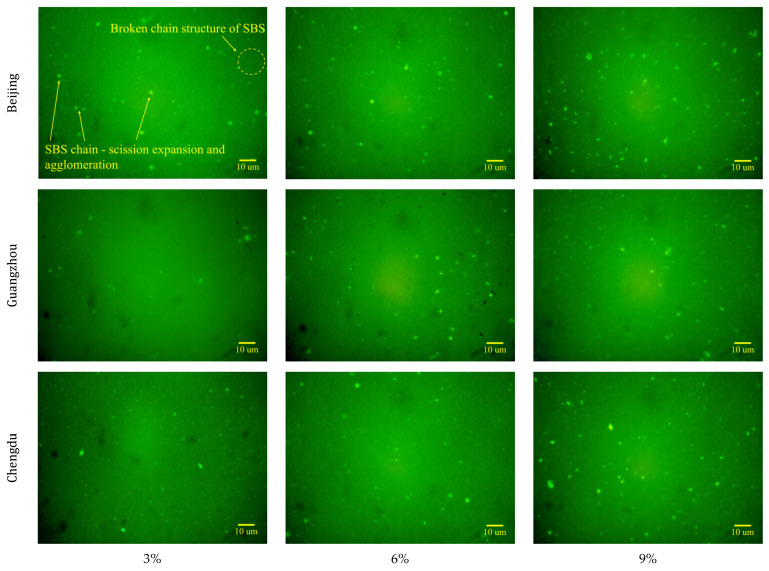
Morphology and distribution of SBS modifiers in samples from Beijing, Guangzhou, and Chengdu under varying contents of industrial animal oil (3%, 6%, and 9%).

The distribution characteristics and variations in SBS in the asphalt binders are illustrated in Figure 10 and Figure 11, respectively. These observations were made under different ageing conditions and following industrial animal oil regeneration, using a microscope with 400× magnification.

As illustrated in Figure 10, unaged asphalt Figure 10a displays a continuous cross-linked network structure of SBS polymers, which substantially enhances the viscosity and cohesion of the asphalt. This structure configuration serves as a fundamental mechanism for maintaining stability and ensuring superior performance. However, following thermal-oxidative ageing Figure 10b, a significant portion of the cross-linked network undergoes thermal-oxidative degradation, resulting in the formation of flocculent and fragmented chain structures. This structural evolution results from two primary mechanisms: (i) the volatilisation of light components (e.g., saturates and aromatics), which reduces the compatibility of asphalt with SBS and weakens structural support, and (ii) the thermal-oxidative degradation of butadiene segments, which disrupts the molecular architecture of SBS. This observation aligns with the decline in the butadiene index reported in Section 4.1. Notably, the substantial reduction in the softening point of thermal-oxidative aged asphalt, as documented in Section 3.1, further underscores the critical importance of cross-linked network integrity in maintaining high-temperature performance.

Under coupled environmental ageing conditions, BEC (Figure 10c), HEC (Figure 10d), and SHEC (Figure 10e) exhibited distinct patterns of structural degradation. As depicted in panels (Figure 10c–e), the aged asphalt exhibit a complete disintegration of the original cross-linked network into smaller, dispersed, and fragmented chains, accompanied by a noticeable reduction in SBS fluorescence intensity. Comparative analysis revealed the following: (i) high-temperature and humidness environment conditions (Guangzhou) resulted in the most dispersed SBS fragments, characterised by the smallest particle dimensions and the weakest fluorescence intensity; (ii) benchmark environment conditions (Beijing) exhibited the largest particle dimensions and the most pronounced fluorescence intensity among the fragmented chains; and (iii) the moderately high-temperature and humidness environment conditions (Chengdu) displayed intermediate characteristics. These findings indicate that high-temperature and humid environments exert the most significant degradation effects on SBS. The humid conditions in the SHEC promote chain scission decomposition, while the SBS degradation caused by the high ultraviolet radiation environment in the BEC is comparatively less pronounced. Under ultraviolet radiation, water undergoes photolysis, generating abundant hydroxyl radicals that act as strong oxidants. These radicals attack the C=C double bonds in the butadiene segments of SBS oxidation reactions. The decomposition process leads to the formation of numerous polar groups, ultimately resulting in the breakdown of the SBS structure. Due to the lower humidness conditions in the BEC, this process was leading to relatively improved sizes and connectivity of SBS chain scission structures. The extent of the aforementioned degradation effects was correlated with the changes in the butadiene index described in Section 4.1. This correlation that the combined effects of high temperature and humidness have a more pronounced impact on the structural degradation of SBS than the effects of increased ultraviolet radiation.

The incorporation of industrial animal oil significantly influenced the microstructural and performance characteristics of aged asphalt. As illustrated in Figure 11, the fragmented SBS chains become miscible with the light components introduced by the industrial animal oil, leading to a gradual attenuation of the fluorescence intensity. Simultaneously, the residual SBS fragments swell and coalesce upon absorbing light components, forming prominent fluorescent light spots. These microstructural transformations were macroscopically manifested as reduced softening points and enhanced ductility, indicating improved material plasticity. With increasing industrial animal oil content, a significant proliferation of fluorescent, enlarged SBS aggregates was observed. This phenomenon reflects two primary mechanisms: (i) the replenishment of light components, which enhances asphalt-SBS compatibility, and (ii) the facilitation of SBS fragment coalescence and swelling. These mechanisms are consistent with the trend of BI elevation documented in Section 4.1.

Comparative analysis of the SBS distribution under various ageing conditions revealed sparse and unevenly distributed fluorescent aggregates under Guangzhou, whereas Beijing produced the most abundant and uniformly distributed fluorescent aggregates. Notably, industrial animal oil was unable to reconstruct fractured SBS molecular chains or restore the cross-linked network architecture. As a result, rejuvenated asphalt exhibits significant performance recovery disparities between high- and low-temperature properties. While low-temperature performance improves, the addition of industrial animal oil fails to compensate for losses in high-temperature performance and, in some cases, even accelerates their decline. These findings underscore the necessity of maintaining strict control over the high-temperature stability of industrial animal oil-rejuvenated asphalt in practical pavement engineering applications to ensure satisfactory road service performance.

## 5. Conclusions

The ageing and regeneration properties, along with the mechanisms of SBS-modified asphalt under the coupled effects of thermal, ultraviolet, and humid conditions in a hot–wet environment, are investigated in this study. The main conclusions are as follows:

- Thermal-oxidative ageing results in the deterioration of asphalt performance, and the severity of ageing is further amplified under thermal-ultraviolet-humidness coupling conditions due to increased humidness and temperature. This is evidenced by the observation that the extent of ageing under high-temperature and humidness environmental conditions is greater than that observed under slightly high-temperature and humidness conditions, both of which are more severe than those observed under benchmark environmental conditions. A comparison between the experimental conditions of slightly high-temperature and humidness environmental conditions (Chengdu) and the benchmark environmental conditions (Beijing) revealed that humidness intensifies the effects of coupled ageing.
- The types of functional groups present in the SBS-modified asphalt remained unchanged under coupled ageing conditions. However, the intensity of the absorption peak associated with butadiene fracture or crosslinking (966 cm⁻^1^) decreased, while the intensities of the absorption peaks corresponding to oxidation (1030 cm⁻^1^) and aromatic conjugation (1600 cm⁻^1^) increased. Following thermal-oxidative ageing, the butadiene index of the asphalt decreased, whereas the sulfoxide and aromaticity indices increased. This process is accompanied by the breakdown of the cross-linked network, a structural feature critical for maintaining performance retention. Thermal-ultraviolet-humidness coupling ageing intensifies the thermal-oxidative degradation of butadiene, leading to the complete conversion of the cross-linked network into fragmented chain structures. Simultaneously, thermal oxidation induces oxidation reactions in sulphide compounds, resulting in the formation of additional sulfoxide groups that strengthen intermolecular forces. Humidness further amplifies this process, facilitating the transfer of lighter components, ultimately leading to asphalt hardening.
- As the proportion of industrial animal oil increased, the low-temperature performance of the SBS-modified asphalt improved; however, the high-temperature performance did not show a corresponding enhancement. At a 9% concentration, the elongation of the aged asphalt under high-temperature and humidness environmental conditions (Guangzhou) remained below the levels observed in the unaged asphalt. Moreover, the most significant changes in the softening point and ductility occurred at a 4% content, after which the extent of change gradually diminished.
- FTIR analysis confirms the chemical interactions between industrial animal oil and aged asphalt, as evidenced by the appearance of a new carbonyl (C=O) peak at 1725 cm⁻^1^. Simultaneously, the butadiene index increased while the sulfoxide index decreased. The primary function of industrial animal oil is to dissolve and dilute the polar molecules generated during the ageing process while replenishing the light components lost over time. The light components introduced by industrial animal oil facilitate the aggregation and expansion of SBS chain fragments; however, they are ineffective in restoring broken crosslinked chain structure of SBS.
- Under benchmark environmental conditions (Beijing), the dissolution and dilution effects on the oxidation products in asphalt are most pronounced, resulting in a noticeable recovery of broken chains. However, as the degree of oxidation in asphalt increases under slightly high-temperature and humidness environmental conditions (Chengdu) and high-temperature and humidness environmental conditions (Guangzhou), the ability of industrial animal oil to mitigate oxidation products is diminished, further exacerbating SBS chain breakage.

## Figures and Tables

**Table 1:** Performance indicators of SBS-modified asphalt.

Technical Indicators	Unit	Prescribed Standards	Experimental Results
Penetration (25 °C, 100 g, 5 s)	mm	40~60	57
Softening Point	°C	>75	82.5
Ductility at 5 °C	cm	>20	61.4
Elastic Recovery (25 °C)	%	≥75	92
Kinematic Viscosity at 135 °C	Pa·S	<3	2.710
Flash Point (Open Cup)	°C	>230	329
Solubility in Trichloroethylene	%	>99	99.74
Density at 15 °C	g/cm^3^	Actual measurement	1.033

**Table 2:** Performance indicators of industrial animal oil.

Technical Indicators	Indicators
Appearance	Light yellow
Kinematic Viscosity at 40 °C, mm^2^/s	30–50
Open Flash Point, °C	260
Density at 20 °C	0.95

**Table 3:** Urban Rainfall Amount.

City	Total Summer Rainfall (mm)	Average Daily Rainfall (mm)
Beijing	6862.80	3.8
Guangzhou	17,556.03	9.7
Chengdu	13,773.7	7.6

**Table 4:** Ultraviolet irradiation time.

City	The Average Total Solar Radiation in Summer (MJ/m^2^)	Ultraviolet Radiation Intensity (MJ/m^2^)
Beijing	1755.6–2048.2	95.095
Guangzhou	1254–1504.8	68.97
Chengdu	1003.2–1254	56.43

**Table 5:** Coupling ageing conditions.

City	Humidness (mm)	Temperature (°C)	Ultraviolet Duration (h)
Beijing	3.8	65	110
Guangzhou	9.7	70	80
Chengdu	7.6	65	65

**Table 6:** The performance of SBS-modified asphalt pavement after ageing.

Ageing Conditions	Softening Point (°C)	Ductility (cm)
Beijing	65.9	20.9
Guangzhou	67.7	16.5
Chengdu	66.4	19.8
Thermal-oxidative ageing	62.4	43.9

**Table 7:** Road performance of SBS-modified asphalt at varying modifier contents.

Ageing Conditions	Softening Point (°C)			Ductility (cm)		
	3%	6%	9%	3%	6%	9%
Beijing	61.4	57.3	54.8	42.3	57.3	63.1
Guangzhou	63.9	60.0	57.3	36.1	48.3	54.6
Chengdu	62.3	58.5	55.8	40.7	54.9	61.7
Thermal-oxidative ageing	57.4	53.5	51.3	58.1	71.4	81.5

**Table 8:** BI, SI, and CI under various ageing conditions.

Ageing Conditions	Butadiene Index	Sulfoxide Index	Aromaticity Index
Beijing	0.1658	0.5077	1.6991
Guangzhou	0.1396	0.5554	1.8442
Chengdu	0.1559	0.5267	1.7651
Thermal-oxidative ageing	0.2227	0.4261	1.3830
Not aged	0.2952	0.3605	1.2453

**Table 9:** BI, SI, and CI values for the conditions of Beijing, Guangzhou, and Chengdu with 6% and 9% industrial animal oil contents.

Ageing Conditions	Butadiene Index		Sulfoxide Index		Aromaticity Index	
	6%	9%	6%	9%	6%	9%
Beijing	0.1834	0.1912	0.3103	0.2936	0.6155	0.5347
Guangzhou	0.1503	0.1538	0.3513	0.3386	0.6908	0.6353
Chengdu	0.1714	0.1770	0.3279	0.3122	0.6512	0.5784

## Data Availability

The original contributions presented in this study are included in the article. Further inquiries can be directed to the corresponding author.
